# The association of exogenous dietary antioxidant micronutrient intake and consumption timing with urinary albumin excretion among U.S. adults

**DOI:** 10.3389/fimmu.2025.1607456

**Published:** 2025-09-23

**Authors:** Hangxu Li, Jingping Ge, Wenhui Tong, Pengpeng Chen, Pan Sun, Sujue Lu, Li Sun, Yang Chen, Yangyang Li, Jun Wang, Jianfei Li, Lantian Mao, Zizhi Li, Xuefeng Jin

**Affiliations:** ^1^ Department of Urology, The Third Affiliated Hospital of Jinzhou Medical University, Jinzhou Medical University, Jinzhou, Liaoning, China; ^2^ Department of Urology, Affiliated Taikang Xianlin Drum Tower Hospital, Medical School of Nanjing University, Nanjing, Jiangsu, China; ^3^ Department of Urology, Taikang Xianlin Drum Tower Hospital Clinical College of Wuhan University, Nanjing, Jiangsu, China; ^4^ Department of Urology, Jinling Hospital, Affiliated Hospital of Nanjing University Medical School, Nanjing, Jiangsu, China; ^5^ Department of Clinical Medicine, Medical College of Yangzhou University, Yangzhou, Jiangsu, China; ^6^ Department of Clinical Medicine, Medical College of Soochow University, Suzhou, Jiangsu, China; ^7^ Shaanxi University of Traditional Chinese Medicine, School of Medicine, Xianyang, Shanxi, China; ^8^ Department of Nursing, Medical College of Nantong University, Nantong, Jiangsu, China; ^9^ Department of Clinical Medicine, Medical College of Jiangsu University, Zhenjiang, Jiangsu, China; ^10^ School of Medicine, Jiangnan University, Wuxi, Jiangsu, China; ^11^ Department of Clinical Medicine, School of Medicine, Xuzhou Medical University, Xuzhou, Jiangsu, China; ^12^ Department of Soft Tissue Surgery, The First Affiliated Hospital of Jinzhou Medical University, Jinzhou, Liaoning, China; ^13^ College of Integrative Medicine, Nanjing University of Chinese Medicine, Nanjing, Jiangsu, China

**Keywords:** exogenous dietary antioxidant micronutrient, meal timing, UAE, albuminuria, NHANES

## Abstract

**Background:**

Oxidative stress plays a central role in the pathogenesis of chronic kidney disease (CKD) and is closely linked to glomerular injury and microvascular endothelial dysfunction. Urinary albumin excretion (UAE) is a sensitive early marker of renal damage and systemic inflammation. Although dietary antioxidants are recognized to modulate oxidative stress, the impact of both their cumulative intake and timing on UAE remains unclear.

**Objectives:**

To investigate the association between the Composite Dietary Antioxidant Index (CDAI)—including both total daily intake and intake at different meals—and the incidence of elevated UAE among adults in the United States. We also aimed to evaluate whether the timing of antioxidant intake, particularly in the evening, modifies this relationship.

**Methods:**

We analysed data from 23,214 adults aged ≥20 years in the U.S. National Health and Nutrition Examination Survey (NHANES) 2009–2018. CDAI was determined using dietary intakes of six antioxidants (vitamins E, A, C, carotenoids, selenium, and zinc) across breakfast, lunch, and dinner. UAE was defined as a urinary albumin-to-creatinine ratio (ACR) >30 mg/g. Weighted multivariable logistic regression, restricted cubic spline analysis, component-independent effect analysis, and analysis of subgroups were used to evaluate the associations and interactions.

**Results:**

Higher CDAI was greatly connected to reduced odds of UAE (fully adjusted OR per SD increase: 0.98; 95% CI: 0.97–0.99; *P* = 0.041). Antioxidant intake during dinner showed the strongest inverse association with UAE (*P* < 0.01), while breakfast and lunch intake were not significantly related. The difference between dinner and breakfast CDAI (ΔCDAI) was also inversely associated with UAE. Subgroup analysis revealed effect modification by BMI: the protective association was attenuated in participants with obesity (BMI ≥ 30).

**Conclusions:**

Both the quantity and timing of dietary antioxidant intake are associated with urinary albumin excretion. Evening antioxidant consumption and a higher ΔCDAI may offer enhanced renal protection, potentially via circadian modulation of oxidative stress and inflammation. These findings support a chrononutrition-based approach to kidney health and warrant further interventional studies.

## 1 Introduction

In addition to being a sign of early kidney illness, elevated urine albumin excretion(UAE) has been demonstrated to be a reliable indicator of the course of cardiovascular risk and chronic kidney disease (CKD) (1–3). The efficiency and ease of the albumin-to-creatinine ratio (ACR) of random urine make it a popular tool for assessing and characterising albuminuria (4, 5). Abnormal elevations in ACR are known to occur at a threshold of 30 mg/g. Albuminuria not only reflects glomerular injury but is also regarded as an early marker of systemic endothelial dysfunction, characterized by increased vascular permeability and impaired barrier integrity (6). According to reports, moderate and/or severe albuminuria (More than 30, less than 300 mg/g) affects 5–19% of the general population; in people with hypertension, the percentage increases to 23%, and in people with diabetes, it can reach 40% (7). Therefore, according to guideline recommendations, patients with diabetes or hypertension should undergo screening once a year. Proteinuria has grown to be a serious public health issue because of its high prevalence and substantial detrimental influence on unfavourable clinical outcomes (8–10).

Consequently, albuminuria should be given high clinical priority. Currently, clinical treatment strategies primarily focus on reducing urinary albumin excretion and delaying the progression of kidney disease (11). Conventional approaches include lifestyle interventions—such as proper dietary control, weight management, and regular physical activity (12–14)—as well as pharmacological treatments, including renin-angiotensin-aldosterone system (RAAS) inhibitors (15), sodium-glucose cotransporter 2 inhibitors (SGLT2i) (16), and mineralocorticoid receptor antagonists (MRAs) (17). In recent years, as understanding of the underlying pathophysiological mechanisms has advanced, emerging therapies such as anti-inflammatory and antioxidant treatments have gained increasing attention, with agents like endothelin receptor antagonists currently undergoing clinical trials. Among these, antioxidant therapy is being actively explored for its potential renoprotective effects (18). Furthermore, precision medicine strategies based on individual risk profiles are considered a promising direction for future interventions to reduce urinary albumin excretion and slow the progression of chronic kidney disease (19).

Oxidative stress plays a central role in the pathogenesis of CKD and is closely linked to both glomerular injury and systemic vascular damage. Beyond its impact on renal function, oxidative stress is a key contributor to cardiovascular diseases, which represent a leading cause of mortality as kidney function deteriorates. Multiple studies have demonstrated that increased oxidative stress accelerates endothelial dysfunction, promotes atherosclerosis, and exacerbates hypertension in individuals with CKD. These interrelated pathways highlight the importance of targeting oxidative stress not only for renal protection but also for cardiovascular risk reduction.

The emergence of novel therapies targeting inflammation and oxidative stress, along with precision medicine approaches based on individual risk profiles, holds great promise in the management of proteinuria. Consequently, there remains an urgent need for reliable and easily applicable indicators to assess the risk of urinary albumin excretion (UAE). To date, numerous studies have demonstrated that the intake of various exogenous dietary antioxidant micronutrients—such as vitamin C, vitamin E, carotenoids, and selenium—is associated with urinary protein excretion, suggesting their potential role as modifiable factors in preventing or managing proteinuria (20–22). However, some conflicting reports indicate that individual dietary antioxidant nutrients may be unrelated to urinary outcomes or may even exert adverse effects. According to a prior study, oral vitamin A supplementation may cause oxidative stress, reduce antioxidant capacity, interfere with redox homeostasis, and make rats more prone to inflammation—all of which run counter to vitamin A’s well-established beneficial antioxidant function (23). The contradictory results imply that isolated chemicals might not be useful in treating oxidative stress-related illnesses (24). Wright et al. developed the Composite Dietary Antioxidant Index (CDAI), a comprehensive metric that accounts for a variety of dietary antioxidants and represents a person’s total antioxidant consumption (25, 26). Based on the combined effect of these nutrients on anti-inflammatory responses, CDAI targets pro-inflammatory indicators, including interleukin-1 beta (IL-1β) and tumour necrosis factor-alpha (TNF-α). These biomarkers are linked to a number of health consequences, such as depression, hypertension, coronary heart disease, cancers, and all-cause mortality (24, 27–30).

Recent research has indicated that the time of meals may affect metabolic and physiological processes, possibly because of the effects of circadian rhythms (31). A main clock in the hypothalamic suprachiasmatic nucleus (SCN) controls the circadian timing system. It communicates with other peripheral clocks, including those in the liver, via neuroendocrine pathways. The light-dark cycle mostly controls the master pacemaker in the SCN, although the liver’s circadian clock is responsive to dietary habits (32). Indeed, dietary patterns may exert direct regulation over the circadian phase of liver function, independently of the SCN and light–dark signalling pathways (33, 34). Therefore, altering eating habits and dietary composition may be a practical way to treat UAE by lowering the body’s levels of oxidative stress. Numerous studies have demonstrated that controlling metabolic status and body weight is significantly influenced by the distribution of vitamins, macronutrients, and minerals throughout the day (35–37). The intricate interplay between the circadian clock and metabolic processes underscores the critical role of meal timing in regulating metabolism (38).

Given these findings, we hypothesize that the intake of exogenous dietary antioxidant micronutrients, as well as the timing of food consumption, may be closely associated with urinary albumin excretion (UAE). This study explored the associations between urinary albumin excretion (UAE) and both daily CDAI levels—measured at breakfast, lunch, dinner, and in total—as well as the intra-day difference in CDAI (ΔCDAI, defined as dinner minus breakfast), using data from participants in the U.S. National Health and Nutrition Examination Survey (NHANES).

## 2 Resources and procedures

### 2.1 Sources of information

Making use of a sample of the US civilian population that is not institutionalized, NHANES is a thorough, Stratified, clustered, and multistage probability survey that represents the whole country. The Centres for Disease Control and Prevention’s Institutional Review Board (IRB) has authorised the poll, guaranteeing that ethical guidelines are followed (30). All information and records pertaining to the research are publicly available. Information permission was gained from all subjects through completed consent forms.

Since 1999, NHANES has collected data annually from approximately 5,000 individuals. The survey is conducted in two-year cycles and includes data from questionnaires, laboratory tests, physical examinations, and sociodemographic information. Detailed information about the program is available on its official website.

### 2.2 Study population

**Figure 1:**
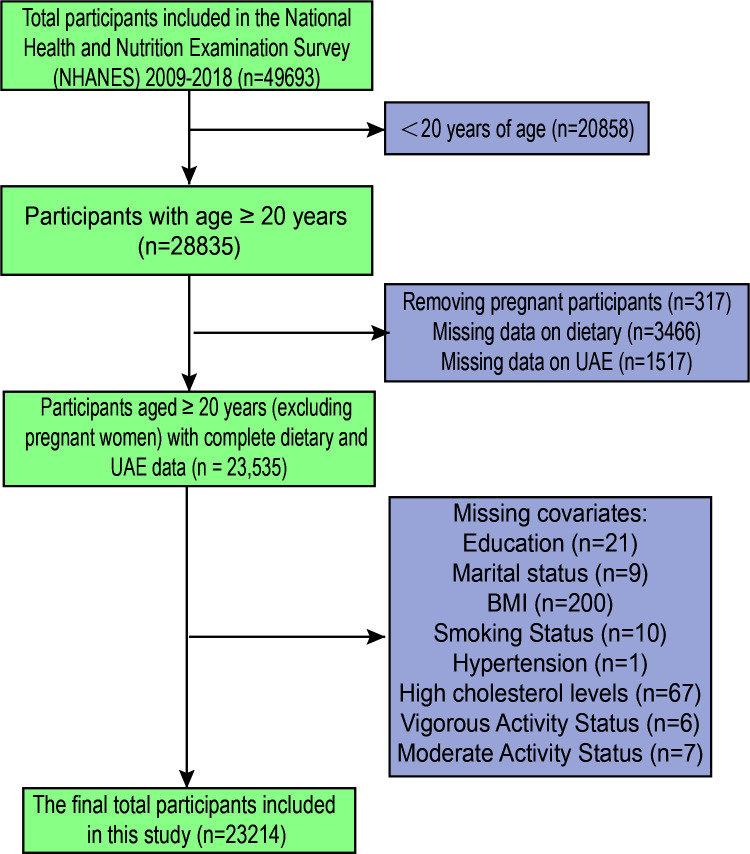
Research participants screening flowchart.

Five-cycle data (2009–2018) from NHANES were analysed, involving 49,693 participants (**Figure 1**). Participants under 20 years of age were excluded to align with common practice in NHANES-based studies focusing on adult populations, and because key variables related to UAE and dietary assessments were more consistently available for individuals aged 20 years and older. We excluded Participants under the age of twenty (n=20,858). Other factors for exclusion were: (1) Pregnant participants (317); (2) lacking information regarding dietary (n=3466); (3) missing UAE data (n=1517); (4) lacking status of education (n=21); (5) unknown marital status (n=9); (6) lacking data on BMI (n=200); (7) lack of smoking status (n=10); (8) lacking data on hypertension (n=1); (9) No data on high cholesterol levels (67); (10) No data on physical activity (13). After the screening, 23,214 participants were included in this study.

### 2.3 Measurement of exogenous dietary antioxidant micronutrient intake

The 24-hour Interview on dietary recall is the present component of nutritional assessment in NHANES. Trained Interviewers for diets, proficient in English and Spanish, conducted the in-person interviews. NHANES collects participants’ dietary intake data using two non-consecutive 24-hour dietary recalls. The initial interview is conducted in a Mobile Examination Center (MEC), where each center includes a dietary interview room equipped with standardized measurement protocols. The second dietary recall is conducted by telephone three to ten days after the first interview.

To reduce bias and improve the data’ dependability, we averaged two measurements. CDAI for each participant was calculated using a modified version developed in previous studies (25). The six dietary antioxidants that were part of CDAI were carotenoids, zinc, selenium, and vitamins E, C, and A. Each micronutrient was standardised by calculating its Z-score, which was obtained by subtracting the mean intake from the individual value and dividing by the overall standard deviation. The Z-scores of the six nutrients were then summed up to generate the composite CDAI value. The following is the formula for the calculation:


$$CDAI=\sum_{i=1}^{n=6}(Individual\;Intake-Mean)/SD$$

Based on when the meals and macronutrients were consumed, the variable “Name of eating occasion” separated them into breakfast, lunch, and dinner. The event label that was used by the responder to describe the eating events influenced the decisions made about their classification. This method provides thorough definitions that consider various cultural customs and social norms (36). The exposure variables in this study included the meal timing of CDAI (total, break-fast, lunch, and dinner) and the difference between dinner CDAI and breakfast CDAI (Δ =dinner − breakfast).

### 2.4 Evaluation of UAE

NHANES participants provided blood and urine samples at standardised Mobile Examination Centers (MECs), and the modified Jaffe kinetic technique and solid-phase fluorescence immunoassay were used to measure urinary albumin and creatinine, respectively. Urine samples for UACR measurement were obtained as random spot samples during MEC visits. The albumin-to-creatinine ratio (ACR) was calculated by dividing the urinary albumin concentration (mg) by the urinary creatinine concentration (g). Increased urinary albumin excretion (albuminuria) was defined as ACR > 30 mg/g (39, 40). Albuminuria was regarded as an outcome variable in our study.

### 2.5 Evaluation of covariates

Based on prior research, we selected potential confounders that may affect UAE, including gender, age, race, education, BMI (Body Mass Index), marital status, PIR (the ratio of family income to poverty), total cholesterol levels, alcohol use, smoking, diabetes, hypertension, status of exercise, eGFR (Estimated Glomerular Filtration Rate), ALT (Alanine Aminotransferase), AST (Aspartate Aminotransferase), triglyceride, creatinine, and uric acid (41–51).

Urinary albumin, urinary creatinine, ALT, AST, triglycerides, serum creatinine, and uric acid were treated as continuous variables. Gender, race, education, and marital status were considered as categorical variables. PIR was categorized into two groups: less than 2 and greater than or equal to 2. BMI was categorized into three groups: less than 25, 25 to less than 30, and 30 or greater. Patients were identified as smokers if they responded “yes” to the question (SMQ020) on smoking. Alcohol use was determined using a drinking question (ALQ130), identifying people as drinkers if they drank 12 or more drinks annually and as non-drinkers if they drank less than 12.

Individuals were classified as having hypertension if their average systolic blood pressure from three readings was ≥140 mmHg, diastolic blood pressure was ≥90 mmHg, they were taking antihypertensive medication, or they answered “yes” to being diagnosed with hypertension. Diabetes was identified based on a “yes” response to a diabetes diagnosis, the use of glucose-lowering medication or insulin, or meeting diagnostic thresholds for glycosylated haemoglobin (≥6.5%) and fasting blood glucose (≥126 mg/dl). There were two categories for total cholesterol levels: low (<240 mg/dl) and high (≥240 mg/dl). Previously told by a doctor that they had hypercholesterolemia or were on lipid-lowering medication, they were considered to have a hypercholesterolemic state, otherwise, they were considered to not be associated with hypercholesterolemia.

### 2.6 Analysis of statistics

During data processing, NHANES sample weights were applied to ensure national representation of the study population. Categorical variables were analyzed using weighted percentages (%) with a chi-square test, while using weighted linear regression, continuous variables were compared, and the results were shown as mean (± SD).

First, we conducted a population description based on CDAI quartiles and performed logistic regression analysis under three models for comparison. Smooth curve fitting and RCS analysis were then applied. Subsequently, a threshold effect analysis was carried out to examine the relationship between CDAI and UAE, and a generalised additive model (GAM) was employed to verify the link between dose and response along with multivariable logistic regression. We then performed multivariable logistic regression models and employed them to assess the correlations of morning, midday, evening, and ΔCDAI with UAE across different models. Independent effect analyses were also conducted for each component of CDAI. In addition, subgroup analyses were performed to explore the stratified associations between CDAI and UAE, and interaction tests were conducted to strengthen the robustness of the results.

To account for multiple comparisons, particularly in component-specific and subgroup analyses, we applied the Benjamini-Hochberg false discovery rate (FDR) correction. An FDR-adjusted p-value of <0.05 was considered statistically significant.

Every statistical analysis was carried out with R (version 4.4.0), with statistical significance characterized by a two-sided p-value < 0.05.

## 3 Result

### 3.1 Features of the population

From NHANES 2009–2018, 23214 suitable participants were chosen based on the screening criteria (**Figure 1**). 2,782 individuals were diagnosed with UAE, while 20,432 individuals did not have UAE. The study population’s baseline characteristics are represented by weighted estimates in **Table 1**. Compared to participants in the first to third quartiles, those in the highest CDAI quartile were more likely to be younger, male, highly educated, non-smokers, physically active (vigorous or moderate), and without hypertension or diabetes. Additionally, the prevalence of UAE decreased as CDAI levels increased. Notable variations in the baseline attributes were also observed across CDAI quartiles in Regarding PIR, marital status, ethnicity, BMI, alcohol consumption, cholesterol levels, eGFR, urinary protein, urinary creatinine, ALT, AST, triglycerides, serum creatinine, and uric acid.

**Table 1:** Weighted fundamental characteristics of participants who were screened (N=23214).

Characteristics	Overall (n = 23214)	CDAI				P value
		Q1 (n=5804)	Q2 (n=5803)	Q3 (n=5803)	Q4 (n=5804)	
Albuminuria						<0.001
Normal	20432 (88.02%)	4954 (85.35%)	5071 (87.39%)	5184 (89.33%)	5223 (89.99%)	
Increased	2782 (11.98%)	850 (14.65%)	732 (12.61%)	619 (10.67%)	581 (10.01%)	
Gender						<0.001
Male	11442 (49.29%)	2071 (35.68%)	2551 (43.96%)	3045 (52.47%)	3775 (65.04%)	
Female	11772 (50.71%)	3733 (64.32%)	3252 (56.04%)	2758 (47.53%)	2029 (34.96%)	
Age (years)						<0.001
<50	11596 (49.95%)	2718 (46.83%)	2811 (48.44%)	2930 (50.49%)	3137 (54.05%)	
>=50	11618 (50.05%)	3086 (53.17%)	2992 (51.56%)	2873 (49.51%)	2667 (45.95%)	
Race						<0.001
Mexican American	3389 (14.60%)	827 (14.25%)	882 (15.20%)	838 (14.44%)	842 (14.51%)	
Other Hispanic	2391 (10.30%)	655 (11.29%)	608 (10.48%)	593 (10.22%)	535 (9.22%)	
Non-Hispanic white	9530 (41.05%)	2160 (37.22%)	2347 (40.44%)	2553 (43.99%)	2470 (42.56%)	
Non-Hispanic black	4966 (21.39%)	1538 (26.50%)	1251 (21.56%)	1067 (18.39%)	1110 (19.12%)	
Other	2938 (12.66%)	624 (10.75%)	715 (12.32%)	752 (12.96%)	847 (14.59%)	
Education						<0.001
Less than 9th grade	2121 (9.14%)	760 (13.09%)	565 (9.74%)	465 (8.01%)	331 (5.70%)	
9-11th grade	3064 (13.20%)	993 (17.11%)	793 (13.67%)	677 (11.67%)	601 (10.35%)	
High school graduate	5240 (22.57%)	1517 (26.14%)	1322 (22.78%)	1249 (21.52%)	1152 (19.85%)	
Some college or AA degree	7142 (30.77%)	1726 (29.74%)	1823 (31.41%)	1787 (30.79%)	1806 (31.12%)	
College graduate or above	5647 (24.33%)	808 (13.92%)	1300 (22.40%)	1625 (28.00%)	1914 (32.98%)	
Marital status						<0.001
Married	11770 (50.70%)	2560 (44.11%)	2948 (50.80%)	3146 (54.21%)	3116 (53.69%)	
Widowed	1709 (7.36%)	594 (10.23%)	458 (7.89%)	381 (6.57%)	276 (4.76%)	
Divorced	2583 (11.13%)	752 (12.96%)	662 (11.41%)	561 (9.67%)	608 (10.48%)	
Separated	779 (3.36%)	243 (4.19%)	220 (3.79%)	168 (2.90%)	148 (2.55%)	
Never married	4379 (18.86%)	1136 (19.57%)	1036 (17.85%)	1038 (17.89%)	1169 (20.14%)	
Living with partner	1994 (8.59%)	519 (8.94%)	479 (8.25%)	509 (8.77%)	487 (8.39%)	
PIR						<0.001
<2	10189 (48.08%)	3101 (59.04%)	2580 (48.85%)	2279 (42.88%)	2229 (41.72%)	
>=2	11003 (51.92%)	2151 (40.96%)	2702 (51.15%)	3036 (57.12%)	3114 (58.28%)	
BMI (kg/m2)						<0.001
<25	6461 (27.83%)	1560 (26.88%)	1523 (26.25%)	1558 (26.85%)	1820 (31.36%)	
>=25, <30	7572 (32.62%)	1779 (30.65%)	1903 (32.79%)	1936 (33.36%)	1954 (33.67%)	
>=30	9181 (39.55%)	2465 (42.47%)	2377 (40.96%)	2309 (39.79%)	2030 (34.98%)	
Smoking						<0.001
Yes	10189 (43.89%)	2705 (46.61%)	2457 (42.34%)	2558 (44.08%)	2469 (42.54%)	
No	13025 (56.11%)	3099 (53.39%)	3346 (57.66%)	3245 (55.92%)	3335 (57.46%)	
Alcohol use						0.043
No	15539 (97.69%)	3482 (97.48%)	3878 (98.05%)	4049 (97.97%)	4130 (97.25%)	
Yes	368 (2.31%)	90 (2.52%)	77 (1.95%)	84 (2.03%)	117 (2.75%)	
Vigorous activity status						<0.001
Yes	4722 (20.34%)	1051 (18.11%)	1066 (18.37%)	1230 (21.20%)	1375 (23.69%)	
No	18492 (79.66%)	4753 (81.89%)	4737 (81.63%)	4573 (78.80%)	4429 (76.31%)	
Moderate activity status						<0.001
Yes	8697 (37.46%)	1947 (33.55%)	2078 (35.81%)	2261 (38.96%)	2411 (41.54%)	
No	14517 (62.54%)	3857 (66.45%)	3725 (64.19%)	3542 (61.04%)	3393 (58.46%)	
Hypertension						<0.001
No	13184 (56.79%)	3051 (52.57%)	3203 (55.20%)	3394 (58.49%)	3536 (60.92%)	
Yes	10030 (43.21%)	2753 (47.43%)	2600 (44.80%)	2409 (41.51%)	2268 (39.08%)	
Diabetes						<0.001
No	18998 (81.84%)	4572 (78.77%)	4672 (80.51%)	4837 (83.35%)	4917 (84.72%)	
Yes	4216 (18.16%)	1232 (21.23%)	1131 (19.49%)	966 (16.65%)	887 (15.28%)	
High cholesterol						0.002
No	13511 (58.20%)	3400 (58.58%)	3309 (57.02%)	3313 (57.09%)	3489 (60.11%)	
Yes	9703 (41.80%)	2404 (41.42%)	2494 (42.98%)	2490 (42.91%)	2315 (39.89%)	
eGFR (ml/min/1.73 m2)						<0.001
<60	1679 (7.55%)	481 (8.72%)	436 (7.84%)	421 (7.55%)	341 (6.11%)	
>=60, <90	7219 (32.48%)	1587 (28.78%)	1676 (30.13%)	1881 (33.74%)	2075 (37.20%)	
>=90	13331 (59.97%)	3446 (62.50%)	3450 (62.03%)	3273 (58.71%)	3162 (56.69%)	
Albumin, urine (mg/L)	9.28 (0.08)	11.20 (0.20)	9.75 (0.17)	8.51 (0.14)	7.99 (0.14)	**<0.001**
Creatinine, urine (mg/dL)	98.26 (0.48)	102.15 (0.98)	99.54 (0.95)	96.00 (0.82)	95.49 (0.94)	**<0.001**
ALT (U/L)	21.61 (0.07)	19.93 (0.13)	21.13 (0.14)	22.26 (0.14)	23.23 (0.15)	**<0.001**
AST (U/L)	23.30 (0.05)	22.54 (0.11)	22.91 (0.10)	23.49 (0.11)	24.30 (0.11)	**<0.001**
Triglycerides (mg/dL)	124.41 (0.51)	122.73 (0.97)	124.42 (1.02)	126.32 (1.04)	124.19 (1.05)	0.236
Creatinine (mg/dL)	0.86 (0.003)	0.85 (0.003)	0.85 (0.003)	0.86 (0.003)	0.87 (0.003)	**<0.001**
Uric acid (mg/dL)	5.27 (0.01)	5.21 (0.02)	5.23 (0.02)	5.29 (0.02)	5.33 (0.02)	**<0.001**

Continuous variables are presented as mean (SE), and Categorical variables are presented as N (%). CDAI, composite dietary antioxidant index; eGFR, Estimated Glomerular Filtration Rate; BMI, body mass index; PIR, the ratio of family income to poverty; ALT, Alanine Aminotransferase; AST, Aspartate Aminotransferase. P-values less than 0.05 are highlighted in bold to indicate statistical significance.

### 3.2 Relationship between CDAI and UAE

**Figure 2:**
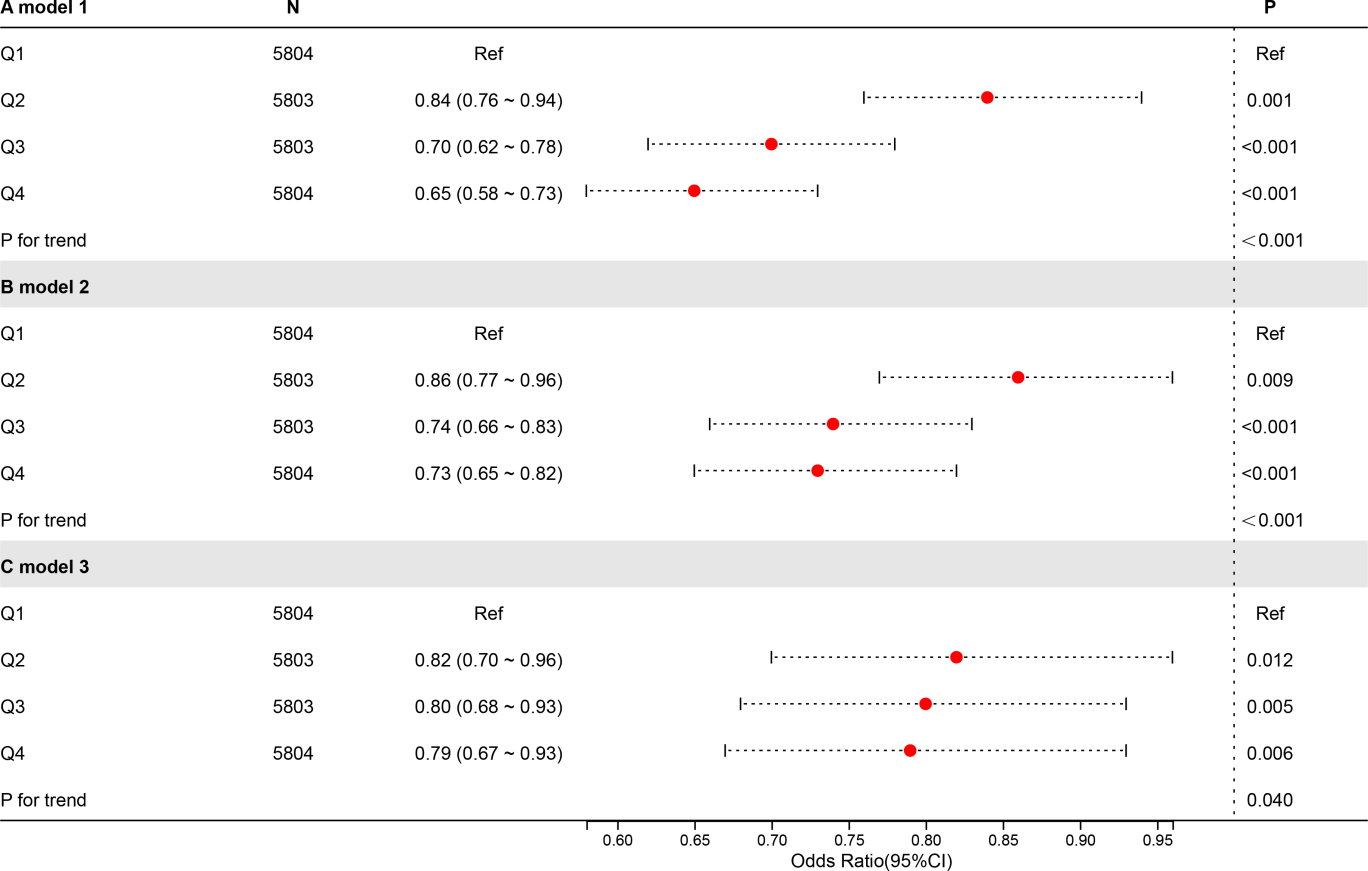
Forest plot of the relationship between CDAI and UAE.

To investigate the connection between CDAI and UAE, an analysis of weighted logistic regression has been carried out using 3 models (**Figure 2**). There were no additional variables in Model 1. In Model 2, age, gender, education, PIR, and race had been considered. Model 3 was constructed utilising Model 2, with modifications made for marital status, diabetes, alcohol use, smoking, hypertension, vigorous activity, moderate activity, eGFR, and total cholesterol level. Quartiles (Q1-4) were created from CDAI; Q1 served as a reference.

Regardless of whether covariates were adjusted for, higher CDAI levels were related to a lower risk of UAE. In Model 3 (totally modified model), the highest CDAI quartile (Q4) was notably negatively connected to UAE contrasted with the lowest quartile (Q1) (OR = 0.79, P = 0.006). Trend tests across all models were significant in terms of statistics (P < 0.05), indicating that the negative connection between higher UAE risk and CDAI is statistically robust.

**Figure 3:**
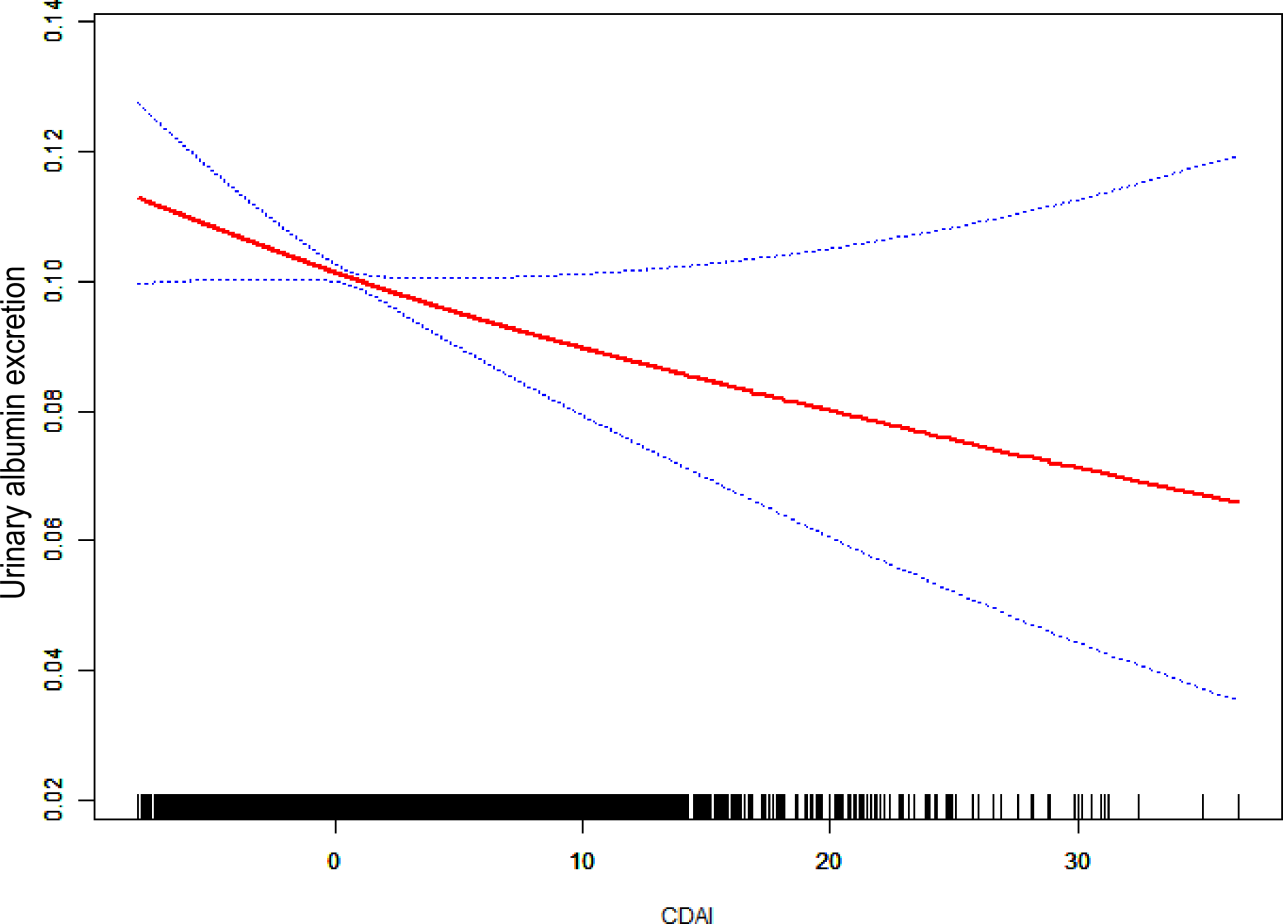
Plotting the link between CDAI and UAE using a smooth curve.

In a completely modified model, the connection between CDAI and UAE was investigated using smoothed curve fitting (**Figure 3**). A substantial negative relationship was identified between UAE and CDAI, indicating that higher dietary antioxidant intake was associated with decreased urinary albumin excretion. The urinary albumin excretion decreased sharply with an increase in CDAI at lower CDAI levels, whereas this decreasing trend became more moderate at higher CDAI values. Additionally, the 95% confidence interval widened considerably at higher CDAI levels, reflecting greater uncertainty in estimating urinary albumin excretion when CDAI was elevated. **Supplementary Figure S1** shows a noteworthy negative correlation has been observed between the CDAI and UAE (P for overall = 0.005), although no significant nonlinearity was detected (P for nonlinear = 0.144). The odds ratio gradually decreased as CDAI increased, indicating a potential protective effect of higher dietary antioxidant intake. Wider confidence intervals at higher CDAI values indicate greater uncertainty.

### 3.3 Dose-response connectivity between CDAI and the UAE

We used an examination of the threshold effect to examine the connection between CDAI and UAE (**Table 2**). In the linear model (Model 1), CDAI was marginally connected with lower odds of albuminuria (OR = 0.986, 95%, *P* = 0.0561). The piecewise regression model (Model two) identified an inflection point at CDAI = 5.476. Below this threshold, CDAI was significantly connected with reduced risk (OR = 0.976, *P* = 0.0209), while no significant association was found above the threshold (OR = 1.007, *P* = 0.6660). The log-likelihood ratio test showed no significant improvement of the threshold model over the linear model (*P* = 0.178).

**Table 2:** The connection between CDAI and albuminuria (analysis of the threshold effect).

	OR (95% CI)	P
Model one		
Linear influence	0.986 (0.972, 1.000)	<0.0561
Model two		
Inflection point (K)	5.476	
CDAI < K	0.976 (0.957, 0.996)	**0.0209**
CDAI > K	1.007 (0.975, 1.041)	0.6660
Log likelihood ratio	0.178	

All analyses were conducted under the completely modified model. P-values less than 0.05 are shown in bold.

**Figure 4:**
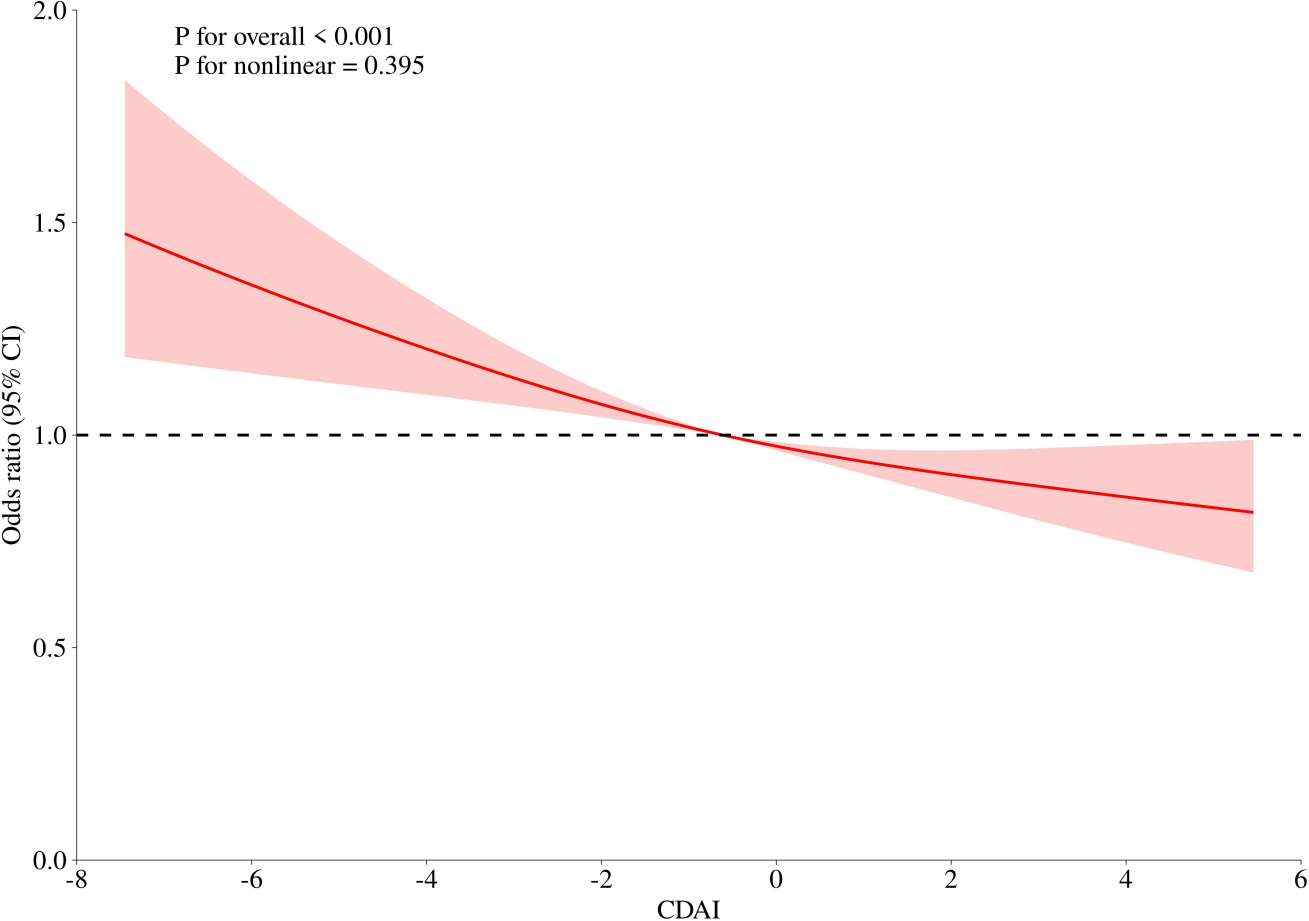
Further dose–response analysis (RCS) of the relationship between CDAI and UAE.

We extracted data from participants with CDAI less than 5.476 and conducted further analysis. As shown in **Figure 4**, the odds ratio for the UAE decreased linearly with increasing CDAI (P < 0.001; P for nonlinearity = 0.395), indicating a significant inverse linear relationship. Higher dietary antioxidant levels were associated with reduced odds of UAE prevalence.

### 3.4 Logistic regression analysis of CDAI and UAE

In the multivariable logistic regression analysis (**Table 3**), CDAI was significantly associated with reduced odds of albuminuria after accounting for every possible confounding factor (OR = 0.98, *P* = 0.041). After applying the FDR correction, the associations between total CDAI, dinner-specific CDAI, and ΔCDAI with UAE remained statistically significant. The corresponding FDR-adjusted p-values are presented in **Supplementary Table S1a**. This indicates that higher dietary antioxidant intake may be protective against albuminuria. In addition, factors such as age, hypertension, diabetes, lower income (PIR), and elevated triglycerides were independently associated with increased risk.

**Table 3:** Multivariable logistic regression analysis for albuminuria.

Variables	OR (95%CI)	*P*
CDAI	0.98 (0.97 ~ 0.99)	**0.041**
Gender (vs male)		
Female	0.99 (0.85 ~ 1.14)	0.864
Age (years)	1.02 (1.01 ~ 1.02)	**<0.001**
Race (vs Mexican American)		
Other Hispanic	0.87 (0.68 ~ 1.11)	0.265
Non-Hispanic white	0.90 (0.74 ~ 1.09)	0.291
Non-Hispanic black	1.06 (0.85 ~ 1.31)	0.610
Other	1.11 (0.87 ~ 1.42)	0.404
Education (vs <9th grade)		
9-11th grade	1.02 (0.78 ~ 1.33)	0.911
High school graduate	0.97 (0.75 ~ 1.25)	0.795
Some college or AA degree	1.01 (0.78 ~ 1.30)	0.950
College graduate or above	0.98 (0.74 ~ 1.29)	0.893
Marital status (vs married)		
Widowed	1.06 (0.84 ~ 1.33)	0.624
Divorced	1.04 (0.87 ~ 1.24)	0.682
Separated	1.19 (0.88 ~ 1.62)	0.263
Never married	1.20 (1.00 ~ 1.44)	0.056
Living with partner	1.05 (0.83 ~ 1.33)	0.691
PIR	0.88 (0.84 ~ 0.91)	**<0.001**
BMI (kg/m2)	1.01 (1.00 ~ 1.01)	0.180
Smoking (vs yes)		
No	0.93 (0.82 ~ 1.05)	0.243
Alcohol use (vs no)		
Yes	1.34 (0.92 ~ 1.94)	0.127
Vigorous activity status (vs yes)		
No	1.03 (0.88 ~ 1.21)	0.704
Moderate activity status (vs yes)		
No	1.05 (0.92 ~ 1.20)	0.433
Hypertension (vs no)		
Yes	1.93 (1.68 ~ 2.21)	**<0.001**
Diabetes (vs no)		
Yes	2.91 (2.54 ~ 3.34)	**<0.001**
High cholesterol (vs no)		
Yes	0.95 (0.83 ~ 1.08)	0.412
eGFR (ml/min/1.73 m2)	1.00 (1.00 ~ 1.00)	0.435
Albumin, urine (mg/L)	1.10 (1.10 ~ 1.11)	**<0.001**
Creatinine, urine (mg/dL)	0.99 (0.99 ~ 0.99)	**0.031**
AST (U/L)	1.01 (1.01 ~ 1.01)	0.050
ALT (U/L)	0.99 (0.99 ~ 1.00)	0.190
Triglycerides (mg/dL)	2.00 (1.03 ~ 3.88)	**0.041**
Creatinine (mg/dL)	1.00 (1.00 ~ 1.00)	0.906
Uric acid (mg/dL)	1.06 (0.98 ~ 1.15)	0.131

The reference group is provided next to the categorical variables. OR values are derived as follows: for continuous variables, each unit increases; for categorical variables, the comparison is made with the reference group. OR, odds ratio; 95% CI, 95% confidence interval. P-values less than 0.05 are shown in bold.

The univariate logistic regression analysis revealed a significant inverse relationship between CDAI and albuminuria (OR = 0.96, *P* < 0.001) (**Supplementary Table S1**). Other factors such as older age, hypertension, diabetes, higher BMI, and elevated triglycerides were positively associated with albuminuria risk, while higher PIR and non-smoking status were protective.

### 3.5 Relationship between UAE and the timing of dietary antioxidant micronutrient intake

**Figure 5:**
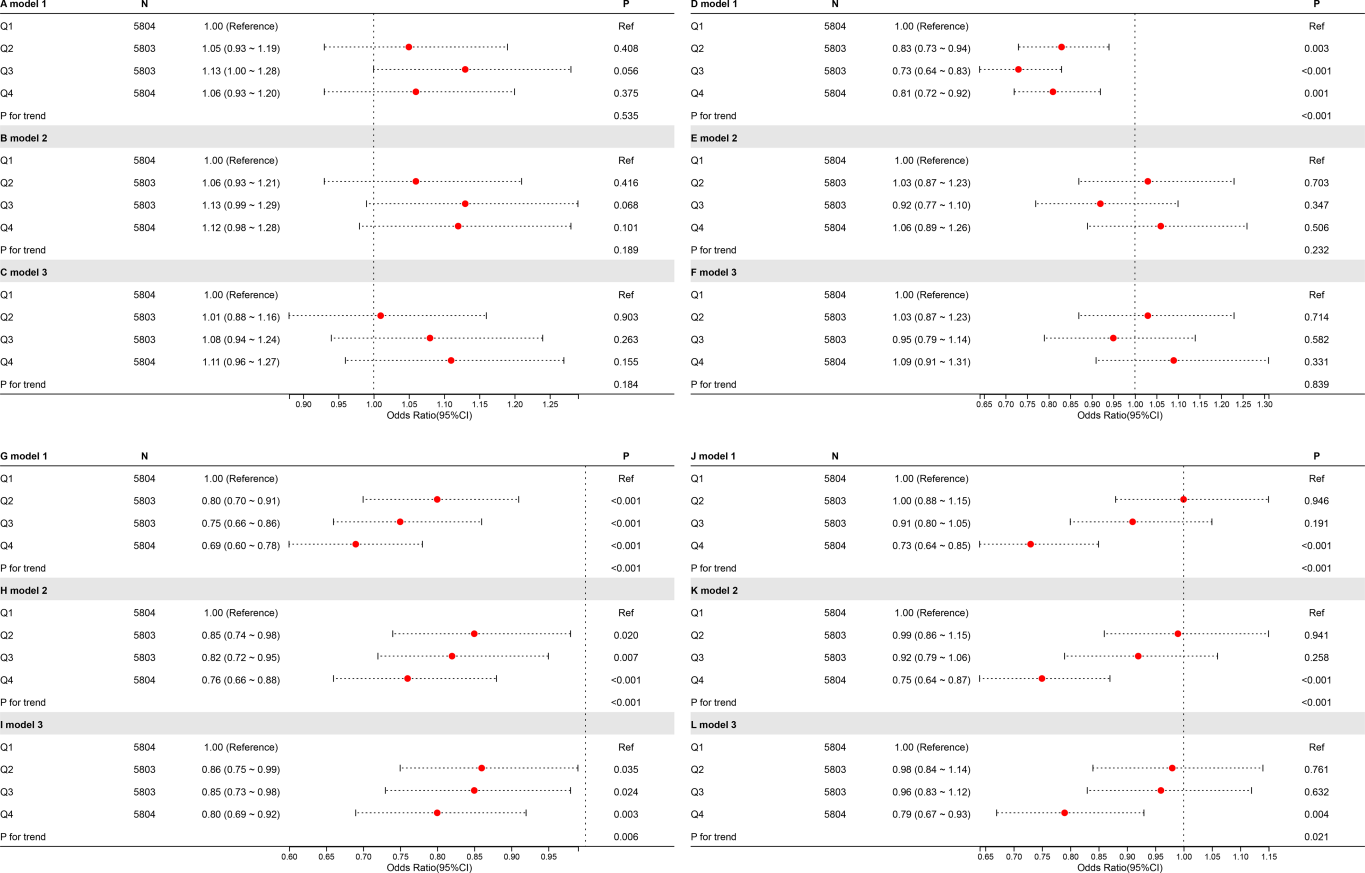
RCS plots of the relationship between meal-specific CDAI and the possibility of albuminuria. The breakfast group is represented by the letters “A,” “B,” and “C”; the lunch group by “D,” “E,” and “F”; the dinner group by “G,” “H,” and “I”; and the Δ group by “J,” “K,” and “L.”.

As shown in **Figure 5**, no significant relationship was observed between CDAI at breakfast or lunch and albuminuria. However, higher CDAI during dinner was substantially linked to a decreased risk of albuminuria. Moreover, the difference between dinner and breakfast CDAI (ΔCDAI) showed a clear inverse relationship with albuminuria, suggesting that antioxidant intake in the evening, rather than the morning, may have stronger protective effects on renal health.

### 3.6 Component-independent impacts of the CDAI

**Figure 6:**
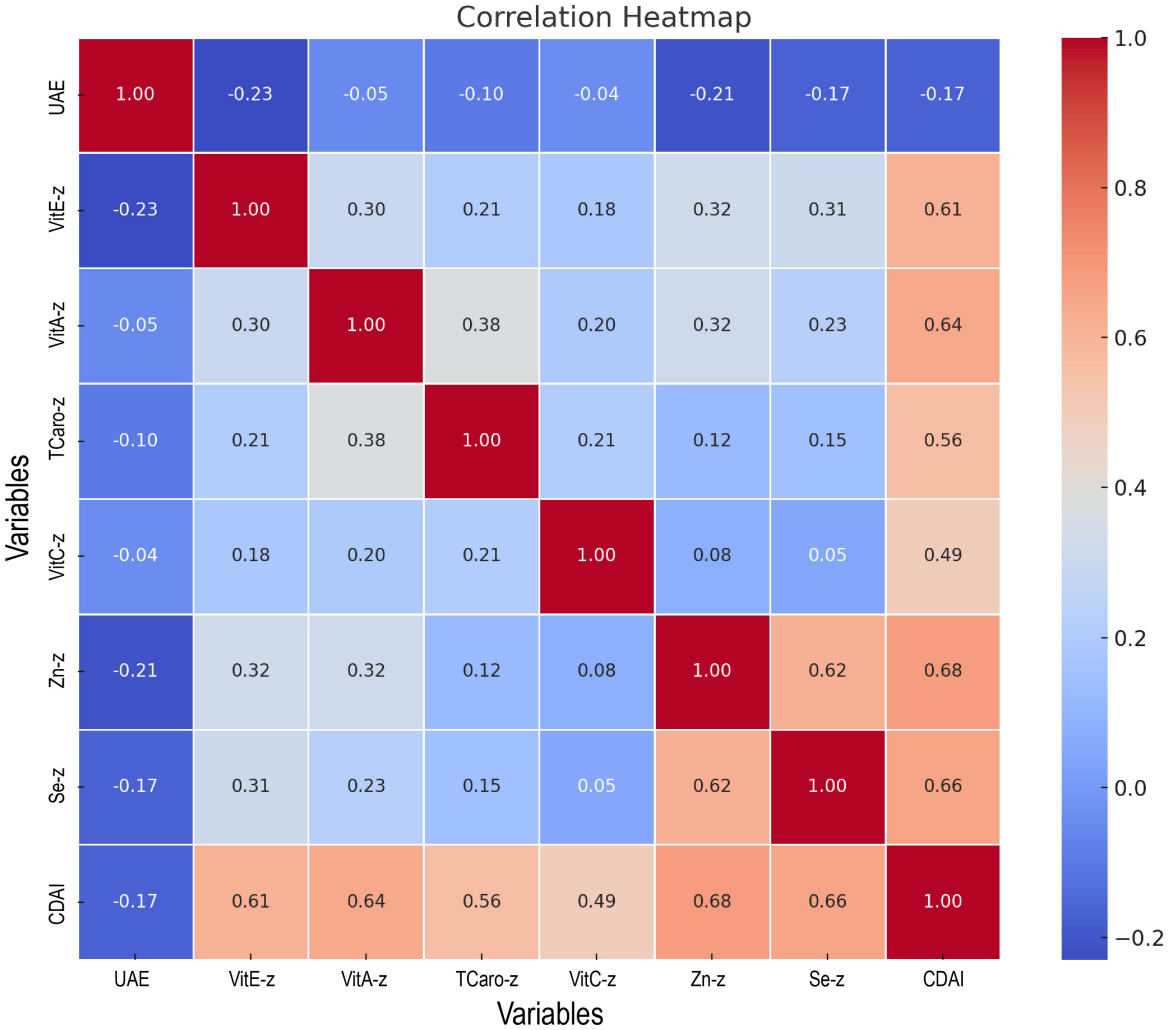
Heatmap of the association between CDAI and its components with urinary albumin excretion.

The six antioxidant elements comprising the CDAI had their Z-score determined. As shown in the correlation heatmap (**Figure 6**), CDAI was moderately and positively correlated with its components, particularly zinc (r = 0.68), selenium (r = 0.66), and vitamin A (r = 0.64), indicating a well-constructed composite index. CDAI was weakly but negatively correlated with urinary albumin excretion (r = -0.17), consistent with earlier findings. Among individual antioxidants, vitamin E (r = -0.23), zinc (r = -0.21), and selenium (r = -0.17) showed the strongest inverse correlations with UAE.

**Figure 7:**
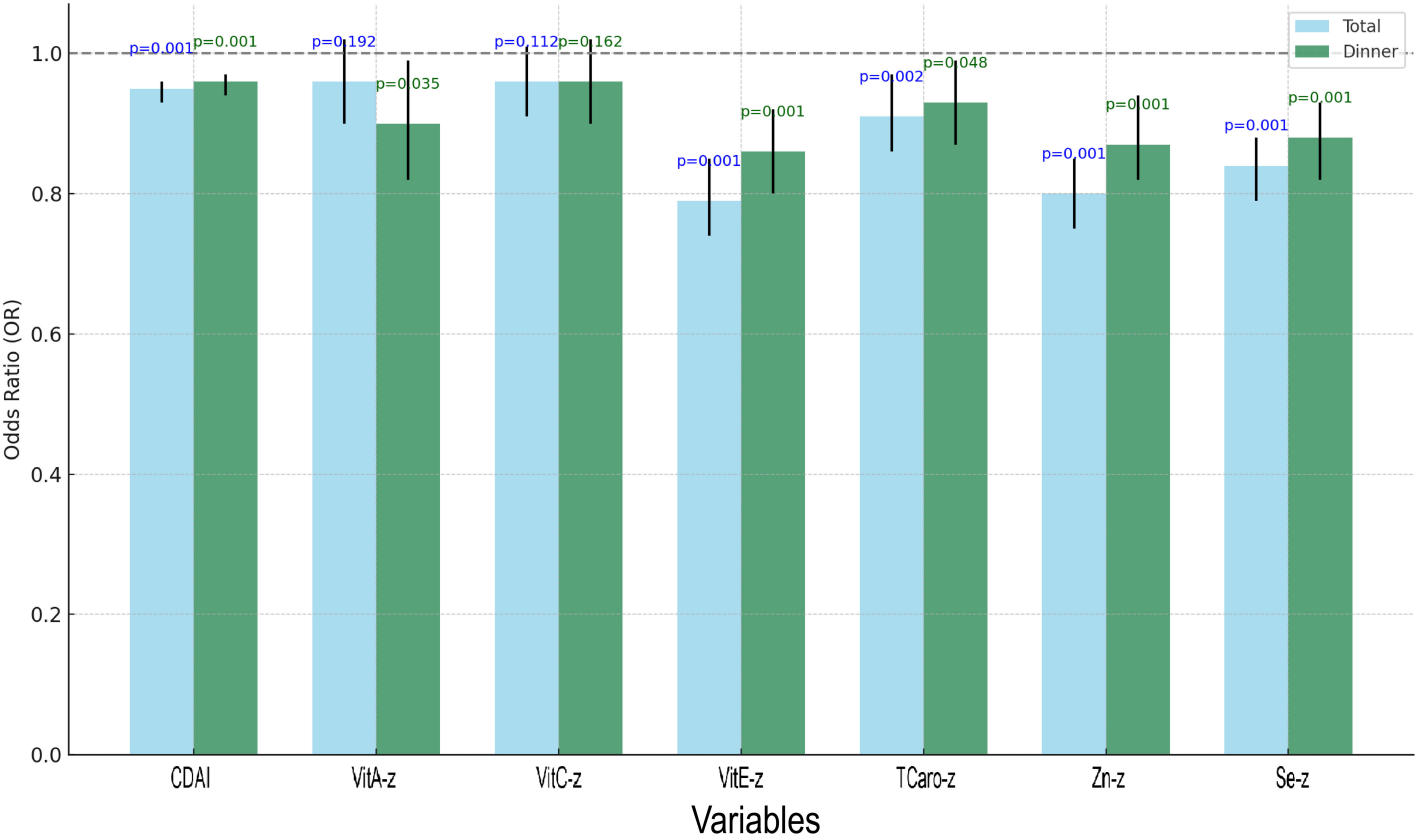
Grouped bar chart of regression analysis for total CDAI, dinner-specific CDAI, and the constituents involved.

Regression analyses of dinner-specific CDAI and total CDAI, along with their components, were conducted under the fully modified model (**Supplementary Table S2**), and the outcomes were visualised (**Figure 7**). Both total and dinner-specific CDAI were substantially linked with reduced odds of albuminuria (P = 0.001 for both). Among the individual components, vitamin E, zinc, and selenium showed consistent and significant protective associations for both total and dinner intake (all P < 0.01). Interestingly, vitamin A was only significant when derived from dinner intake (P = 0.035), while vitamin C was not significantly associated in either model. These results suggest that not only the quantity but also the timing of antioxidant intake may influence renal outcomes.

### 3.7 Assessment of the relationship between CDAI and UAE across subgroups

**Figure 8:**
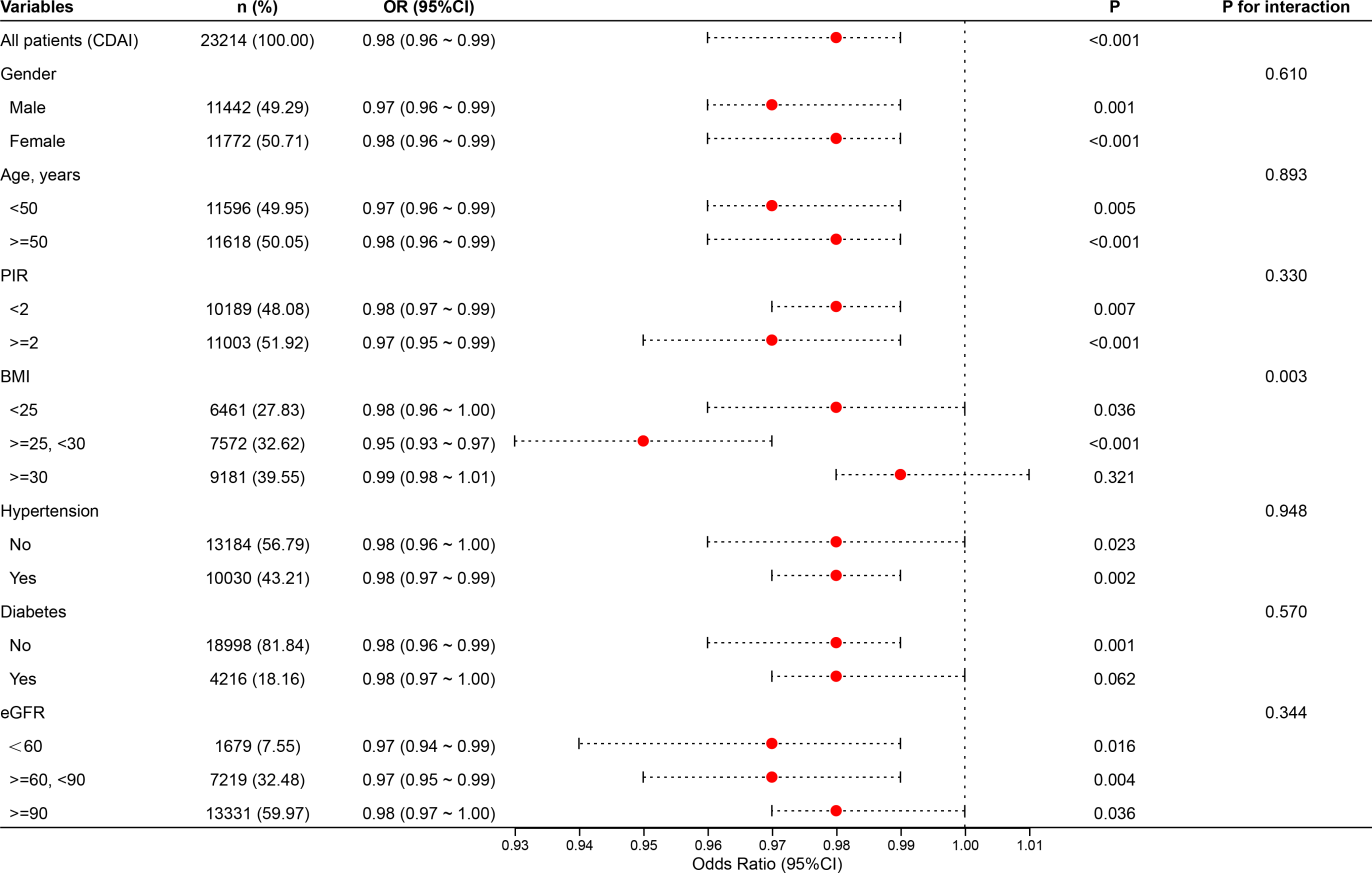
Stratified relationships between CDAI and UAE prevalence.

The study conducted stratified analyses based on sex, age, PIR, BMI, diabetes, hypertension, and eGFR (**Figure 8**). Subgroup analyses revealed that the inverse association between CDAI and albuminuria remained robust across various population strata, including gender, age, poverty-income ratio (PIR), hypertension, diabetes status, and kidney function (eGFR), with all P for interaction values exceeding 0.05. This suggests that the protective effect of dietary antioxidants is generally stable and not modified by these factors. Notably, significant interaction was observed in the BMI subgroup (P for inter = 0.003). The protective relationship between CDAI and albuminuria was evident among individuals with BMI <30, but this association was attenuated and no longer statistically significant among those with BMI ≥30. This finding may imply that obesity could blunt the antioxidant benefit, potentially due to altered oxidative stress pathways, chronic low-grade inflammation, or antioxidant bioavailability in adipose tissue.

Although the interaction by kidney function (eGFR) was not statistically significant (P = 0.344), the consistent inverse associations across all eGFR categories highlight that the renal benefit of higher CDAI persists even among individuals with mildly to moderately impaired kidney function. These results support the potential universal relevance of antioxidant-rich diets for renal protection, regardless of baseline renal status or cardiometabolic conditions.

## 4 Discussion

This study, leveraging a huge, nationally typical NHANES sample, provides novel evidence that higher composite dietary antioxidant intake (CDAI) is associated with a lower risk of urinary albumin excretion (UAE), a key marker of endothelial dysfunction and early renal injury. Importantly, the timing of antioxidant intake emerged as a significant modifier of this relationship—dinner-specific CDAI and ΔCDAI (dinner minus breakfast) showed the most robust inverse associations with UAE. These findings introduce two novel aspects not reported in previous NHANES-based analyses: the identification of dinner-specific antioxidant intake as the most protective timing pattern for reducing UAE, and the introduction of ΔCDAI as a practical and novel indicator reflecting intra-day antioxidant distribution and its relevance to renal health.

**Figure 9:**
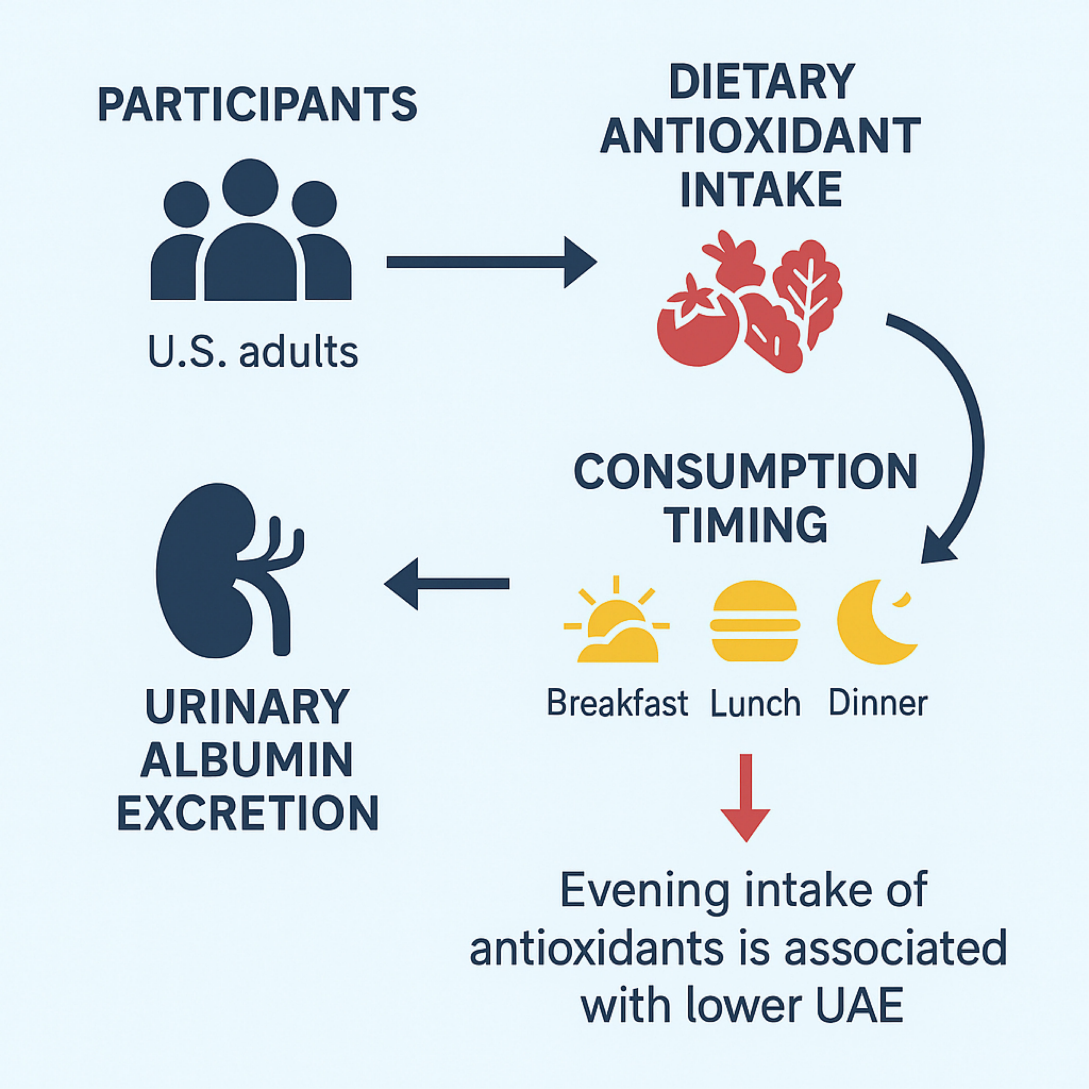
Graphical summary of the study design and key findings.

A graphical summary illustrating the study design and key findings is presented in **Figure 9**.

Prior research investigations have demonstrated that oxidative stress and low-grade inflammation lead to albuminuria via impairment of the glomerular filtration barrier (52). Antioxidant micronutrients such as vitamin E, zinc, and selenium may modulate these pathways by downregulating pro-inflammatory mediators and scavenging reactive oxygen species, including TNF-α and IL-1β—key immune regulators (53–55). While supplementation trials have yielded inconsistent results, likely due to limited synergism, CDAI reflects integrated antioxidant exposure and may thus offer greater explanatory power. Our findings align with emerging interest in diet-induced immune modulation (56). Antioxidant-rich diets may indirectly modulate immune-metabolic cross-talk and endothelial activation, both of which are relevant to subclinical kidney inflammation and albumin leakage (57–59).

Oxidative stress promotes endothelial dysfunction through multiple interconnected mechanisms. Elevated reactive oxygen species (ROS) levels activate inflammatory pathways, disrupt endothelial nitric oxide synthesis, and damage tight junction proteins within the glomerular filtration barrier (60). This cascade leads to increased vascular permeability and subsequent urinary albumin excretion. Targeting oxidative stress may therefore offer dual benefits in mitigating both renal and cardiovascular risks (61).

The stronger association of dinnertime CDAI with UAE reduction suggests that synchronizing antioxidant intake with circadian-regulated immune and oxidative stress responses could enhance renal resilience (62). Feeding-fasting cycles entrain peripheral clocks, particularly in the kidney and liver, which are highly responsive to metabolic cues (63). Evening antioxidant intake may provide timely defense against nocturnal oxidative peaks, enhancing anti-inflammatory responses and protecting the glomerular endothelium (64). ΔCDAI may serve as a practical biomarker for identifying chrononutritional imbalance in antioxidant distribution. Records show that high levels of several important antioxidants in the human body, containing superoxide dismutase (SOD), glutathione peroxidase (GSH-PX), and malondialdehyde (MDA), and melatonin, as well as white blood cell types like neutrophils, monocytes, and lymphocytes, occur at night or shortly before dawn (65). The circadian expression patterns of several antioxidant enzymes, including glutathione reductase (GR), catalase (CAT), and glutathione peroxidase (GPx), have also been demonstrated to be influenced by vitamin A (66).

Subgroup analyses confirmed the protective association across diverse populations; however, the attenuation of antioxidant benefits observed in individuals with obesity (BMI ≥30) may reflect several underlying mechanisms. Obesity is characterized by chronic low-grade inflammation, increased oxidative stress, and altered antioxidant bioavailability. Specifically, excessive adipose tissue may sequester fat-soluble antioxidants such as vitamins A and E, reducing their systemic availability (67, 68). Additionally, obesity-related changes in gut absorption, hepatic metabolism, and circulating lipid profiles can further influence antioxidant distribution and function (69). These factors may collectively diminish the renoprotective effects of dietary antioxidants in obese individuals (70). While these explanations are supported by prior studies, our cross-sectional design and lack of biomarker data preclude direct validation. Future investigations combining dietary assessments with serum antioxidant measurements and inflammatory markers are warranted to clarify these pathways.

This study’s strengths include its large sample size, nationally representative data, and novel focus on both antioxidant quantity and timing. However, several limitations must be considered. First, dietary antioxidant intake was assessed using self-reported 24-hour dietary recalls, which may introduce recall bias and measurement error. Although we applied the average of two non-consecutive 24-hour recalls mitigating variability, this method is inherently limited by participants’ memory and reporting accuracy. Moreover, serum antioxidant biomarkers, which could provide objective measures of antioxidant status, were not consistently available across all NHANES cycles included in our analysis. We have therefore not incorporated biomarker data in this study. Future longitudinal research integrating both detailed dietary assessments and biomarker measurements is warranted to validate and extend our findings, particularly in relation to the timing of antioxidant intake and its physiological effects. Studies integrating dietary patterns with immunophenotyping, circadian transcriptomics, and inflammation biomarkers could clarify the mechanistic underpinnings of these associations and advance precision nutrition approaches in renal-immunometabolic health.

Our findings suggest that in addition to focusing on the amount of antioxidant intake, paying attention to its timing—particularly emphasizing evening consumption—may be important for renal protection. These insights highlight a potential role for personalized chrononutrition strategies in reducing albuminuria risk and improving both renal and cardiovascular outcomes. Although our study is observational, it provides a basis for future interventional research and suggests actionable dietary recommendations that could complement existing clinical practice guidelines.

## 5 Conclusions

This study emphasises the possible contribution of both the quantity and timing of antioxidant micronutrient intake in mitigating urinary albumin excretion among U.S. adults. The observed benefits of evening antioxidant consumption open new avenues for targeted dietary strategies to prevent or manage early kidney damage. These findings may contribute to the development of personalized nutrition interventions aligned with circadian biology to improve renal outcomes.

## Data Availability

The original contributions presented in the study are included in the article/**Supplementary Material**. Further inquiries can be directed to the corresponding authors.
