# Supplementary figures and images for: The association of exogenous dietary antioxidant micronutrient intake and consumption timing with urinary albumin excretion among U.S. adults
Source: Front Immunol. 2025 Sep 23;16:1607456. doi: 10.3389/fimmu.2025.1607456 (PMC12500451; doi:10.3389/fimmu.2025.1607456)

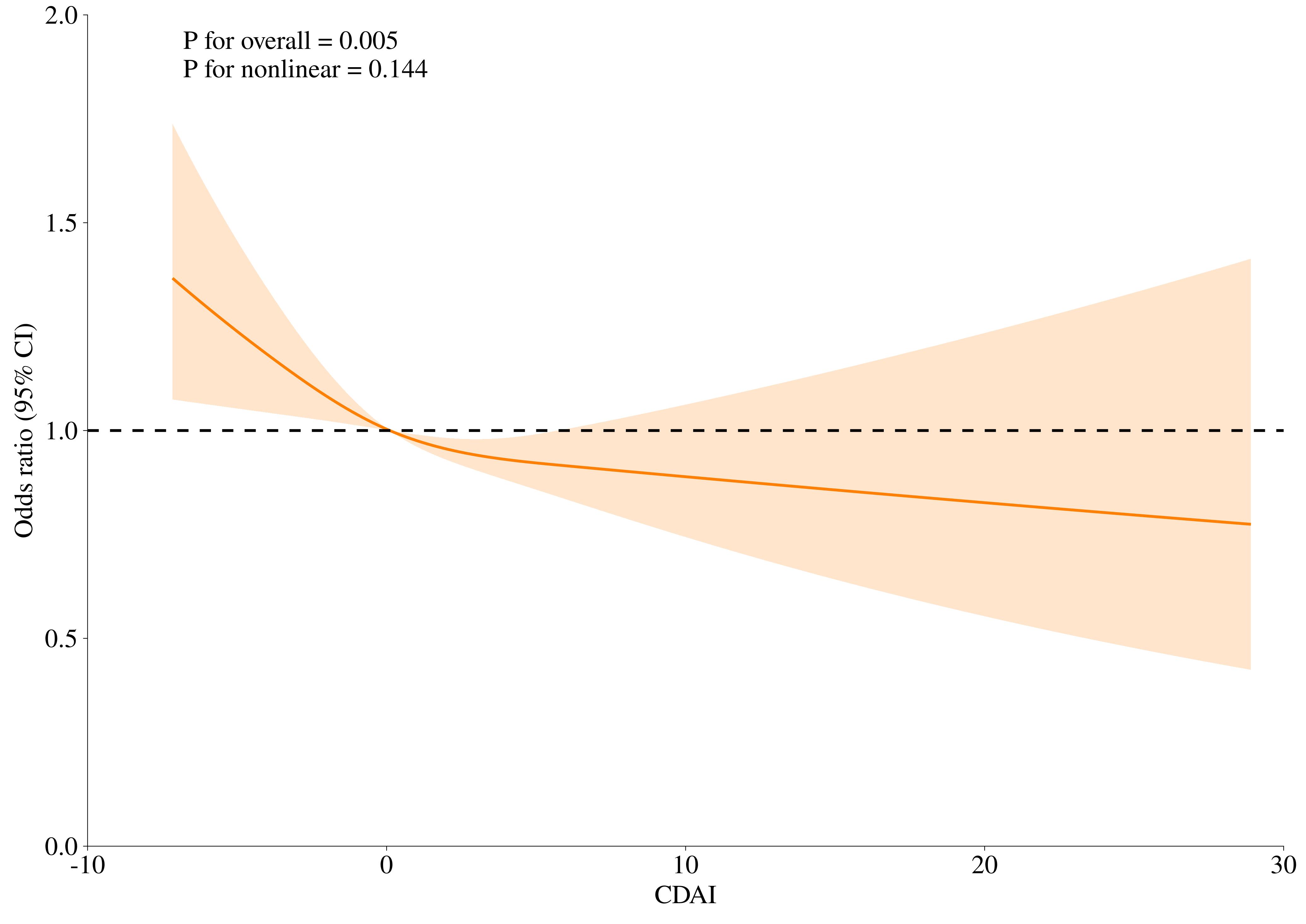

Supplement: Supplementary file 1 [file Image1.tiff]
