# Supplementary material for: The association of exogenous dietary antioxidant micronutrient intake and consumption timing with urinary albumin excretion among U.S. adults
Source: Front Immunol. 2025 Sep 23;16:1607456. doi: 10.3389/fimmu.2025.1607456 (PMC12500451; doi:10.3389/fimmu.2025.1607456)
Supplement: Supplementary file 3 [file Table1.docx]

Table S1 Univariate logistic regression analysis for albuminuria

| Variables | OR (95%CI) | *P* |
| --- | --- | --- |
| CDAI | 0.96 (0.95 ~ 0.97) | **<0.001** |
| Gender |  |  |
| Male | 1.00 (Reference) |  |
| Female | 1.00 (0.92 ~ 1.08) | 0.975 |
| Age, years | 1.04 (1.03 ~ 1.04) | **<0.001** |
| Race |  |  |
| Mexican American | 1.00 (Reference) |  |
| Other Hispanic | 0.90 (0.77 ~ 1.06) | 0.220 |
| Non-Hispanic white | 0.87 (0.77 ~ 0.98) | **0.020** |
| Non-Hispanic black | 1.12 (0.99 ~ 1.28) | 0.075 |
| Other | 0.83 (0.71 ~ 0.97) | **0.017** |
| Education |  |  |
| Less than 9th grade | 1.00 (Reference) |  |
| 9-11th grade | 0.72 (0.62 ~ 0.83) | **<0.001** |
| High school graduate | 0.61 (0.53 ~ 0.70) | **<0.001** |
| Some college or AA degree | 0.55 (0.48 ~ 0.63) | **<0.001** |
| College graduate or above | 0.40 (0.35 ~ 0.46) | **<0.001** |
| Marital status |  |  |
| Married | 1.00 (Reference) |  |
| Widowed | 2.36 (2.08 ~ 2.67) | **<0.001** |
| Divorced | 1.22 (1.07 ~ 1.38) | **0.002** |
| Separated | 1.27 (1.03 ~ 1.57) | **0.023** |
| Never married | 0.73 (0.65 ~ 0.82) | **<0.001** |
| Living with partner | 0.75 (0.63 ~ 0.88) | **<0.001** |
| PIR | 0.86 (0.83 ~ 0.88) | **<0.001** |
| BMI | 1.02 (1.02 ~ 1.03) | **<0.001** |
| Smoking |  |  |
| Yes | 1.00 (Reference) |  |
| No | 0.78 (0.72 ~ 0.85) | **<0.001** |
| Alcohol use |  |  |
| No | 1.00 (Reference) |  |
| Yes | 1.13 (0.81 ~ 1.56) | 0.482 |
| Vigorous activity status |  |  |
| Yes | 1.00 (Reference) |  |
| No | 1.37 (1.23 ~ 1.52) | **<0.001** |
| Moderate activity status |  |  |
| Yes | 1.00 (Reference) |  |
| No | 1.30 (1.20 ~ 1.42) | **<0.001** |
| Hypertension |  |  |
| No | 1.00 (Reference) |  |
| Yes | 3.62 (3.32 ~ 3.94) | **<0.001** |
| Diabetes |  |  |
| No | 1.00 (Reference) |  |
| Yes | 4.40 (4.05 ~ 4.79) | **<0.001** |
| High cholesterol |  |  |
| No | 1.00 (Reference) |  |
| Yes | 1.84 (1.70 ~ 1.99) | **<0.001** |
| eGFR | 0.99 (0.99 ~ 0.99) | **<0.001** |
| Albumin, urine (mg/L) | 1.10 (1.10 ~ 1.11) | **<0.001** |
| Creatinine, urine (mg/dL) | 0.99 (0.99 ~ 0.99) | **<0.001** |
| ALT (U/L) | 1.00 (1.00 ~ 1.00) | **0.517** |
| AST (U/L) | 1.01 (1.01 ~ 1.01) | **<0.001** |
| Triglycerides (mg/dL) | 4.04 (3.55 ~ 4.59) | **<0.001** |
| Creatinine (mg/dL) | 1.01 (1.01 ~ 1.01) | **<0.001** |
| Uric acid (mg/dL) | 1.20 (1.17 ~ 1.23) | **<0.001** |

Abbreviations: OR: odds ratio; 95% CI: 95% confidence interval.
