# Supplementary material for: The association of exogenous dietary antioxidant micronutrient intake and consumption timing with urinary albumin excretion among U.S. adults
Source: Front Immunol. 2025 Sep 23;16:1607456. doi: 10.3389/fimmu.2025.1607456 (PMC12500451; doi:10.3389/fimmu.2025.1607456)
Supplement: Supplementary file 4 [file Table2.docx]

|  | Total | | Dinner | |
| --- | --- | --- | --- | --- |
|  | OR (95%CI) | *P* | OR (95%CI) | *P* |
| CDAI | 0.95 (0.93 ~ 0.96) | **<0.001** | 0.96 (0.94 ~ 0.97) | **<0.001** |
| Z-score of VA | 0.96 (0.90 ~ 1.02) | 0.192 | 0.90 (0.82 ~ 0.99) | **0.035** |
| Z-score of VC | 0.96 (0.91 ~ 1.01) | 0.112 | 0.96 (0.90 ~ 1.02) | 0.162 |
| Z-score of VE | 0.79 (0.74 ~ 0.85) | **<0.001** | 0.86 (0.80 ~ 0.92) | **<0.001** |
| Z-score of Carotene | 0.91 (0.86 ~ 0.97) | **0.002** | 0.93 (0.87 ~ 0.99) | **0.048** |
| Z-score of Zn | 0.80 (0.75 ~ 0.85) | **<0.001** | 0.87 (0.82 ~ 0.94) | **<0.001** |
| Z-score of Se | 0.84 (0.79 ~ 0.88) | **<0.001** | 0.88 (0.82 ~ 0.93) | **<0.001** |

Supplementary Table 2. Fully adjusted regression analysis of total CDAI and evening CDAI, along with their individual components.
